# Supplementary material for: The Lack of Analgesic Efficacy of Nefopam after Video-Assisted Thoracoscopic Surgery for Lung Cancer: A Randomized, Single-Blinded, Controlled Trial
Source: J Clin Med. 2022 Aug 18;11(16):4849. doi: 10.3390/jcm11164849 (PMC9409862; doi:10.3390/jcm11164849)
Supplement: Supplementary file 1 [file jcm-11-04849-s001.zip › jcm-1839996-supplementary.pdf]

Table S1. The details of Brief pain inventory-short form on 3 months after surgery

	**Control Group (n = 37)**	**Nefopam Group (n = 38)**	**Median difference 95% CI**	***P* value**
Presence of pain	24 (65)	21 (55)		0.540
Pain intensity[1]				
Worst pain	3.00 [2.00, 4.00]	2.00 [1.00, 3.00]	-1.0 [-2.0, 1.0]	0.150
Least pain	0.00 [0.00, 0.00]	0.00 [0.00, 0.00]	0.0 [0.0, 0.0]	0.189
Average Pain	1.00 [0.75, 2.00]	1.00 [0.00, 2.00]	0.0 [-2.0, 1.0]	0.299
Current Pain	0.00 [0.00, 1.25]	0.00 [0.00, 0.00]	0.0 [-1.0, 0.0]	0.272
Pain interference with[1]				
General activity	0.00 [0.00, 0.00]	0.00 [0.00, 0.00]	0.0 [0.0, 0.0]	0.097
Mood	0.00 [0.00, 0.00]	0.00 [0.00, 0.00]	0.0 [0.0, 0.0]	0.369
Walking ability	0.00 [0.00, 0.00]	0.00 [0.00, 0.00]	0.0 [0.0, 0.0]	0.599
Normal work	0.00 [0.00, 0.00]	0.00 [0.00, 0.00]	0.0 [0.0, 0.0]	0.290
Relations with other people	0.00 [0.00, 0.00]	0.00 [0.00, 0.00]	0.0 [0.0, 0.0]	0.097
Sleep	0.00 [0.00, 0.00]	0.00 [0.00, 0.00]	0.0 [0.0, 0.0]	0.098
Enjoyment of life	0.00 [0.00, 0.00]	0.00 [0.00, 0.00]	0.0 [0.0, 0.0]	0.705

Values are number (proportion) or median [interquartile range]. For median difference, 95% CI are computed by the 2.5th and 97.5th percentiles of the bootstrap distribution by 1000 bootstrap replications. BPI-SF: Brief Pain Intensity-short form, each item except for presence of pain is rated on NRS (from '0' = [no pain] to '10' [worst] or as '0' [no interference] to '10' [interferes completely]).; [1]Pain intensity scores and interference items of BPI-SF were analyzed only among the patients who answered to have current pain (control group, n=24; nefopam group, n= 21).; CI, confidence interval

Table S2. The short form of neuropathic pain questionnaire on 3 months after surgery

	**Control Group (n = 37)**	**Nefopam Group (n = 38)**	**Median difference 95% CI**	***P* value**
Tingling pain	0.0 [0.0, 10.0]	0.0 [0.0, 10.0]	0.0 [-10.0, 10.0]	0.535
Numbness	0.0 [0.0, 0.0]	0.0 [0.0, 0.0]	0.0 [0.0, 0.0]	0.344
Increased pain due to touch constant	0.0 [0.0, 20.0]	0.0 [0.0, 6.0]	0.0 [-20.0, 10.0]	0.153

Values are median [interquartile range]. For median difference, 95% CI are computed by the 2.5th and 97.5th percentiles of the bootstrap distribution by 1000 bootstrap replications.; Each item rated from '0' = (no pain) to '100' (worst); CI, confidence interval
